# Effect of methyl-beta-cyclodextrin on the viability and acrosome damage of sex-sorted sperm in frozen-thawed bovine semen

**DOI:** 10.1186/s40709-016-0043-x

**Published:** 2016-04-12

**Authors:** Seunghyung Lee, Yong-Seung Lee, Sang-Hee Lee, Boo-Keun Yang, Choon-Keun Park

**Affiliations:** College of Animal Life Sciences, Kangwon National University, Chuncheon, 24341 Republic of Korea; Institute of Animal Resources, Kangwon National University, Chuncheon, 24341 Republic of Korea

**Keywords:** Methyl-beta-cyclodextrin, Sex sorting, Viability, Acrosome damage, Spermatozoa

## Abstract

**Background:**

The regulation of methyl-beta-cyclodextrin (MBCD) on cryodamage on X- and Y-sperm during cryopreservation of semen was investigated. The semen was collected from ten healthy bulls of proven fertility by an artificial vagina. The bovine sperm treated with MBCD fresh solution (0, 1, 5, 10, and 20 mM). The sperms were evaluated for viability and acrosome damage using flow cytometry. Moreover, X- and Y-sperm in frozen-thawed bovine semen were sorted by flow cytometry after Hoechst 33342-dyed, and the viability and acrosome damage of sperms were analyzed.

**Results:**

Sperm viability in frozen-thawed semen was decreased by MBCD (*p* < 0.05), also the acrosome damage of sperm was significantly increased (*p* < 0.05). Moreover, we sorted X- and Y-sperm from frozen-thawed bovine semen for observing the viability and acrosome damage on the separated X- and Y-sperm after MBCD treatment. Viability of X-sperm was significantly lower than that of Y-sperm (*p* < 0.05). Also, acrosome damage of X-sperm was significantly higher than Y-sperm (*p* < 0.05).

**Conclusions:**

Methyl-beta-cyclodextrin enhances damage of sperm in frozen-thawed bovine semen, and X-sperm is more sensitive than Y-sperm in cell damage. These results demonstrate that MBCD can inhibit viability of spermatozoa in frozen-thawed bovine semen (for X-sperm, especially).

## Background

The technology of controlling the sex of mammalian offspring is of great importance in the livestock industry [1]. There has been great interest in sex pre-selection in bovine, and gained clear economic benefits and management advantages. Sex is determined by the presence or absence of the Y chromosome in mammals. In the process of spermatogenesis, X- and Y-sperm can be produced, and the sex of the embryo is determined by the chromosome of the sperm. The X-bearing sperm (X-sperm) in bovine is larger and longer than the Y-bearing sperm (Y-sperm), having 3.8 % more DNA [2, 3]. The X-sperm swims more slowly and has a longer life span than Y-sperm [4]. Sorting for X- and Y-sperm populations based on differences in DNA content by flow cytometry was recommended for improving the selection of X- and Y-sperm [5] and it is widely used in livestock animals. Flow cytometry is able to analyze the biological characteristics of several thousand cells in real time using fluorescent dye or fluorescent antibodies and can actively separate and isolate cells having specified properties. The use of flow cytometry also provides a new opportunity to discover differences in biological characteristics between separated populations of X- and Y-sperm [6, 7].

The main function of sperm is to produce embryos through fertilizing the oocyte, and capacitation indicates the fertility of sperm. Therefore, the difference in acrosome reaction between X- and Y-sperm during capacitation is important. Mammalian sperm undergo a series of biochemical transformations in the female reproductive tract that are collectively known as capacitation [8]. The signaling pathways involved in sperm capacitation have been characterized and current evidence indicates that cholesterol efflux, pH, Ca^2+^, actin polymerization, cAMP, and tyrosine phosphorylation are important regulatory components [9–14]. Cholesterol, a major structural constituent of the plasma membrane, plays an important role as a regulator of plasma membrane function [15]. Methyl-beta-cyclodextrin (MBCD), which is a cyclic heptasaccharide consisting of beta gluco-pyranose units effectively extracts sperm sterols, resulting in the disruption of detergent resistant sperm membranes [16, 17].

Therefore, this study investigated the effect of MBCD on acrosome damage and viability of X- and Y-sperm in bovine frozen-thawed semen for improving the selection of X- and Y-sperm.

## Results

### Sperm was damaged by MBCD in frozen-thawed bovine semen

**Fig. 1:**
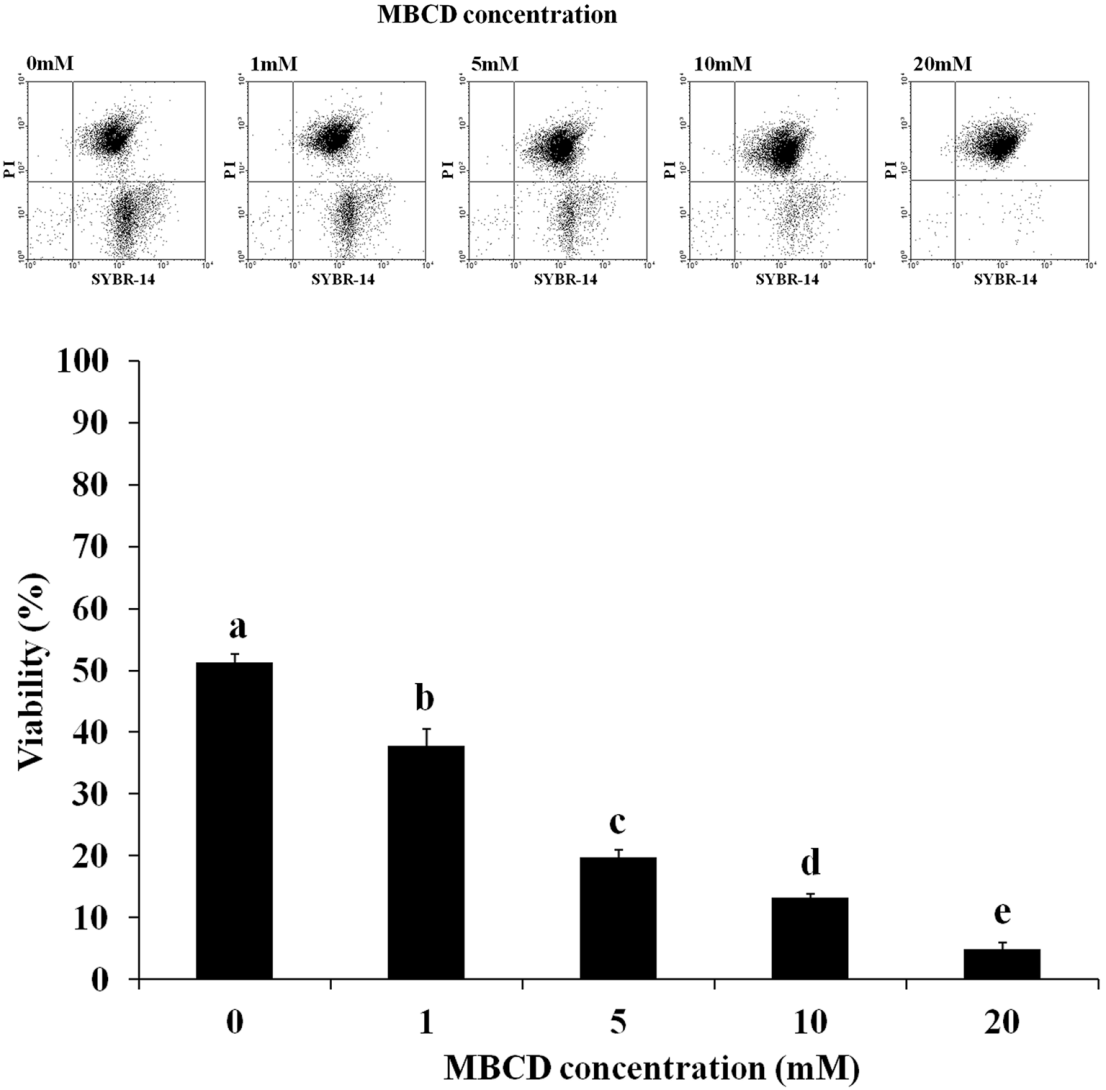
Effect of MBCD on viability of frozen-thawed bovine sperm. After staining with SYBR-14 and PI, the viability of sperm was analyzed by flow cytometry method (fluorescent intensity of SYBR-14 and PI, *top*). Data were appeared as means ± SEM, n = 30 (*p* < 0.05, *bottom*)

**Fig. 2:**
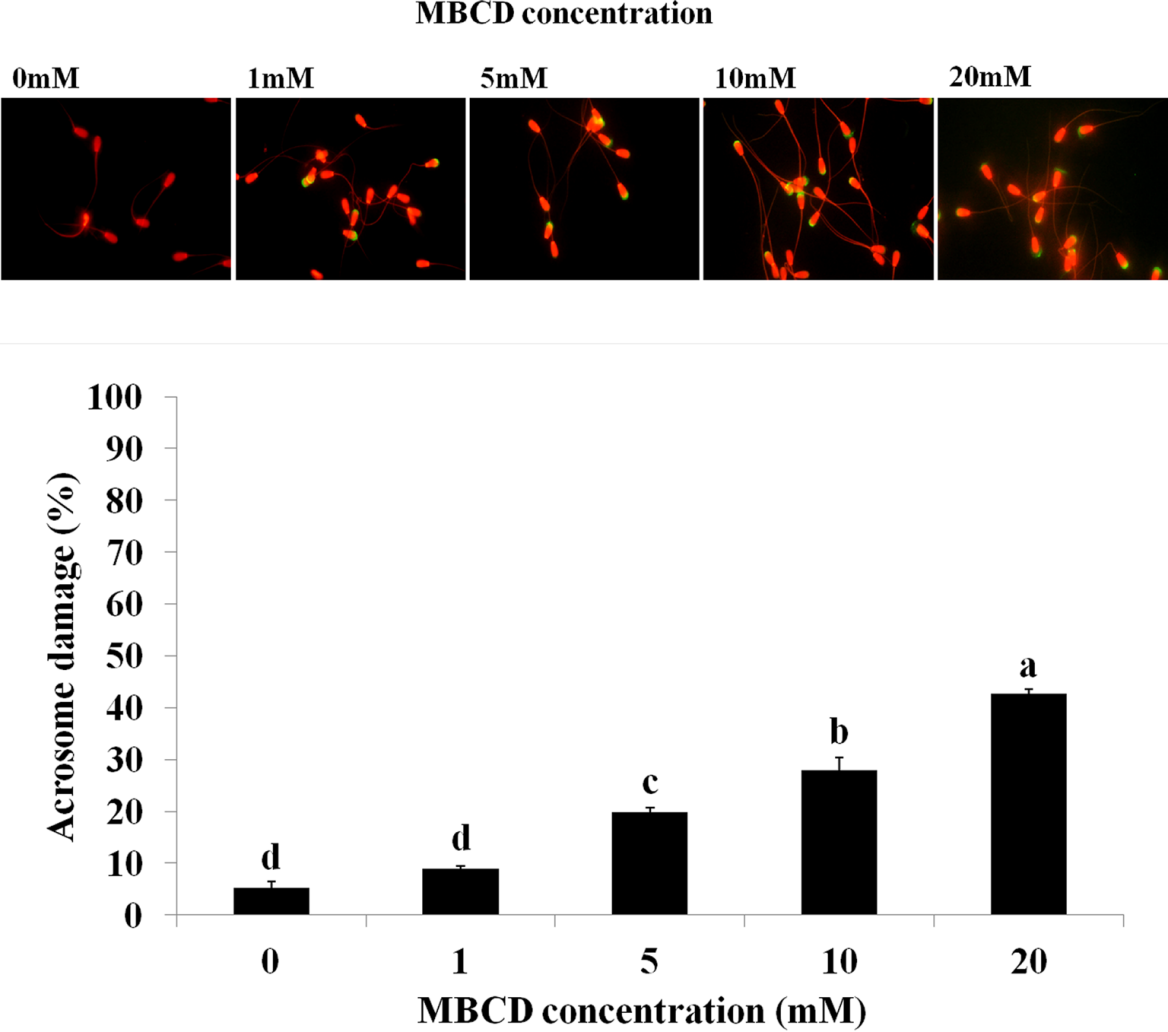
Effect of MBCD on acrosome damage of frozen-thawed bovine sperm. Acrosome damage in sperm was analyzed by flow cytometry, and the staining patterns were verified by inspecting sperm samples under an epifluorescence microscope (*top*). Data were appeared as means ± SEM, n = 30 (*p* < 0.05, *bottom*)

Sperm viability in frozen-thawed bovine semen was decreased by MBCD (Fig. 1, *p* < 0.05). In the first set of experiments, the effect of MBCD on viability was evaluated using different MBCD concentrations (0, 1, 5, 10, and 20 mM). Viability of the treated-MBCD samples were significantly decreased in frozen-thawed sperm (52.2 ± 1.8 %, 37.5 ± 2.1 %, 19.4 ± 1.1 %, 16 ± 0.5 %, and 7.7 ± 0.9, respectively). We also observed sperm acrosome damage after MBCD treatment in all tested concentrations (0, 1, 5, 10, and 20 mM, Fig. 2). In result, acrosome damage was significantly increased by MBCD (6.3 ± 1.1 %, 9.2 ± 0.5 %, 19.4 ± 0.9 %, 28.4 ± 2.8 %, and 45.4 ± 1.0, respectively, *p* < 0.05).

### Effects of MBCD on the viability and acrosome damage of sperm in sex-sorted X- and Y-sperm

**Fig. 3:**
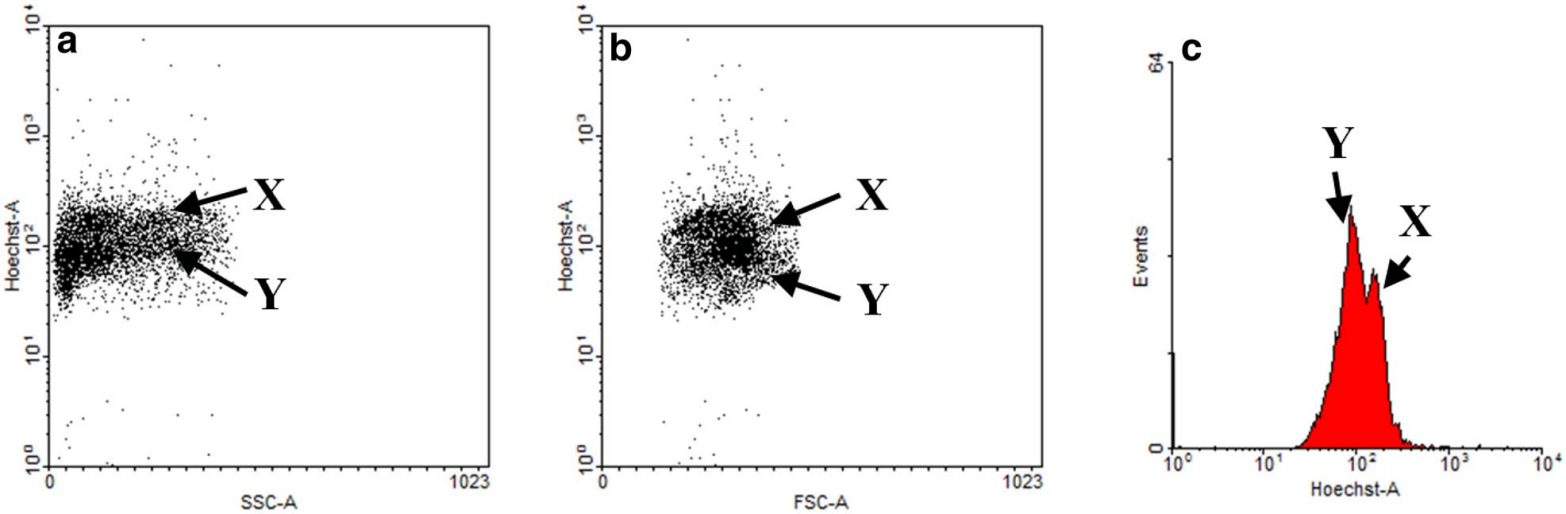
*Dot plot* and histogram of X- and Y-chromosomes after Hoechst 33342 staining for sex sorting in frozen-thawed bovine sperm. Frozen-thawed sperm was automatically sorted into X- and Y-sperm populations by flow cytometry. The sex-sorted sperm based on differences of DNA contents. A *dot plot* is displaying Hoechst 33342 fluorescent intensity versus side-angle scatter (SSC, **a**) and forward angle scatter (FSC, **b**). Histogram is displaying Hoechst 33342 fluorescent intensity of sorted X- and Y-sperm (**c**)

**Fig. 4:**
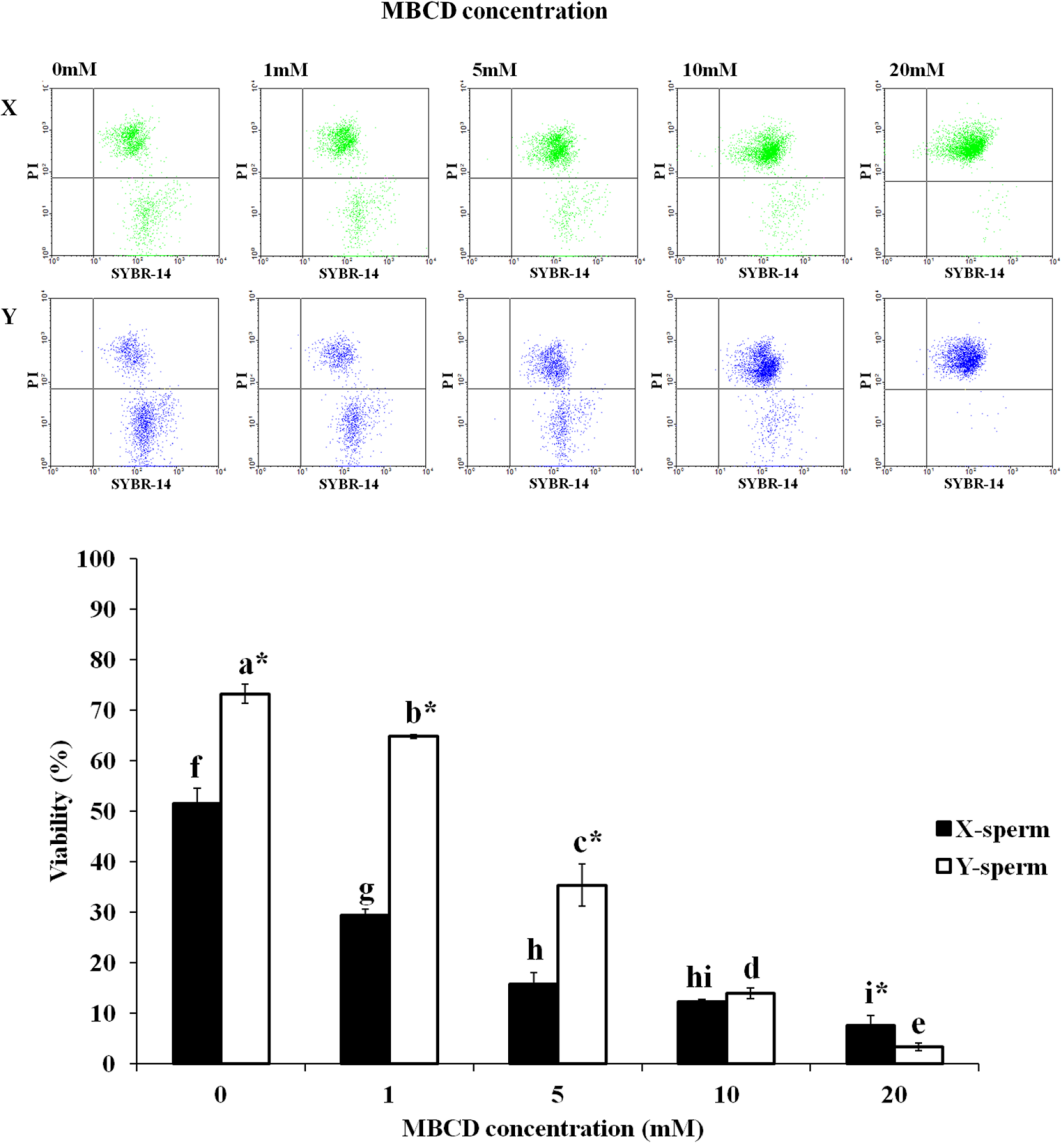
Hoechest 33342-dyed sperms were separated to X and Y populations based on differences of DNA content by flow cytometry. *Green spots* fluorescent intensity in X chromosome sperm. *Blue spots* fluorescent intensity in Y chromosome sperm (*top*). The frozen-thawed X and Y sperm dyed with SYBR-14 and PI, and viability was analyzed by fluorescence intensity in flow cytometric dot plot (*bottom*). **a**–**i** Different letters are significantly different in viability of X (**a**–**e**) and Y (**f**–**i**) sperm (*p* < 0.05). *Different letters are significantly difference in viability between X and Y sperm (*p* < 0.05)

**Fig. 5:**
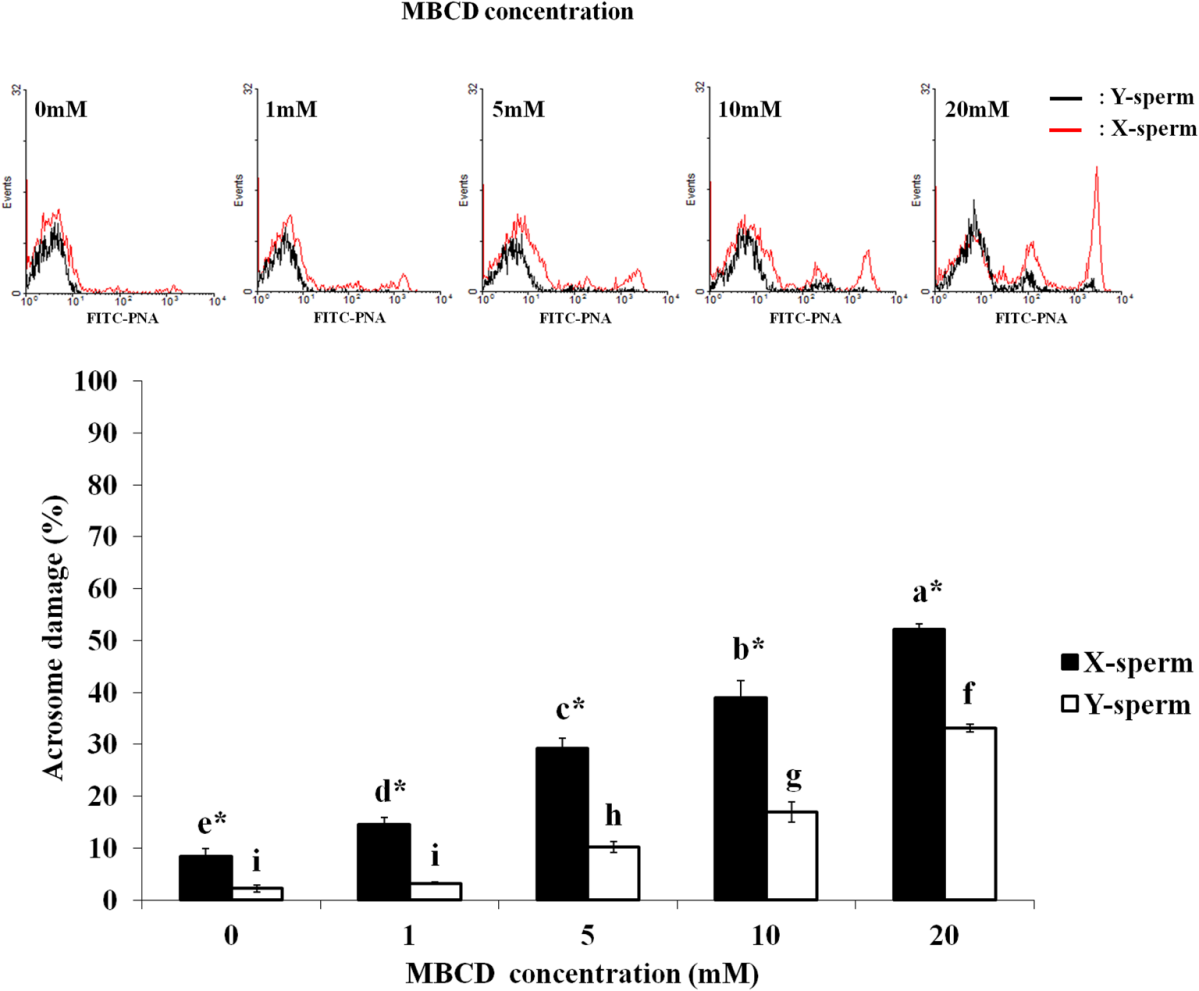
Acrosome damage of X and Y sperm in frozen-thawed semen. Samples dyed with FITC-PNA after MBCD treatment (0, 1, 5, 10, and 20 mM). *Red line* FITC fluorescent intensity in X chromosome sperm. *Black line* FITC fluorescent intensity in Y chromosome sperm (*top*). Acrosome damage was observed by FITC fluorescence intensity using a flow cytometer (*bottom*). **a**–**i** Different letters are significantly different in percentages of acrosome damage after treatment with various MBCD concentrations in X (**a**–**e**) and Y (**f**–**i**) sperm (*p* < 0.05). *Different letters are significantly difference in percentages of acrosome damage in X and Y sperm (*p* < 0.05)

In this experiment, frozen-thawed sperms dyed with Hoechst 33342 were separated to X- and Y-sperm populations based on differences in DNA contents (Fig. 3). As shown on the overlaid histogram, FITC–PNA-dyed X-sperm had a higher fluorescent intensity. Then, the viability and acrosome damage using sorted X- and Y-sperm were evaluated. Y-sperm viability was significantly higher than that of X-sperm on non-treated MBCD (Fig. 4, *p* < 0.05). After frozen-thawed sperms treated with 1 and 5 mM MBCD (73.1 ± 1.7 % and 66.3 ± 0.2, respectively), Y-sperm viability was also higher than X-sperm, but not 10 and 20 mM MBCD (35.4 ± 3.1 % and 14.3 ± 1.0, respectively). Acrosome damage of X-sperms was significantly higher (*p* < 0.05) than Y-sperms in all MBCD concentrations (9.5 ± 1.3 %, 15.2 ± 1.1 %, 29.5 ± 1.9 %, 39.7 ± 3.3 %, and 52.4 ± 0.8 %, respectively—Fig. 5).

## Discussion

We analyzed the difference in membrane damage to the bovine X- and Y-sperm. The method developed by Johnson et al. [18] for sexing sperm on the basis of their DNA content was applied. Our investigation was to test the sexing procedure to Johnson et al. method in cryopreserved sperm. Flow cytometry can sort sperm at >90 % accuracy [5]. In this study, X- and Y-sperm were separated according to Hoechst 33342 intensity by flow cytometry, but the sorting procedure likely causes damage to sperm by laser and sorting stress [2]. Therefore, the multi-fluorescence-stained (Hoechst 33342, FITC–PNA or SYBR-14/PI) sperms were separated into populations of X- and Y-based on differential DNA content. The biological characteristics of sperm were analyzed in each population by multi-analytic ability of flow cytometry.

Methyl-beta-cyclodextrin is very efficient in stimulating the efflux of membrane cholesterol from the sperm cells in vitro [16]. The efflux of cholesterol induces changes in membrane architecture and fluidity, and it thereby facilitates membrane-dependent processes like the acrosome reaction [19]. In this study, increased acrosome damage to sperm after MBCD treatment was observed by FITC-PNA staining (Figs. 3, 4). Lectin FITC-PNA binds exclusively to the outer acrosome membrane of damaged sperm [20]. Also, viability of sperms dyed with SYBR-14 and PI was shown to decrease with MBCD concentrations increased on the flow cytometric dot plot (Figs. 5 and 6). The viability of sperm was decreased, since MBCD damaged sperm membranes.

These results could suggest that acrosome reactions in X-sperm could occur more quickly than in Y-sperm. The X-sperm may have a lower cholesterol/phospholipid ratio in sperm membrane than Y-sperm. It is generally known that MBCD extracts cholesterol from the sperm plasma membrane [21]. The cholesterol decrease in this membrane also plays a role in the induction of the acrosome reaction. The cholesterol/phospholipid ratio of the sperm membrane is a major determinant in membrane fluidity and stability during cryopreservation [22]. Cholesterol reduces the transition temperature of membrane, and maintains them in a fluid state at reduced temperatures, thereby, reducing the membrane damage that occurs at low temperatures [23]. Therefore, X-sperm may be more sensitive to low temperatures than the Y-sperm. In this study, viability of Y-sperm (72.3 ± 1.9 %) in control group without MBCD was significantly higher than that of X-sperm (51.5 ± 3.0 %). These differences were apparently caused by the cholesterol ratio in membrane and sensitivity to cold shock in X- and Y-sperm.

## Conclusion

Bovine sperms suffered acrosome membrane damage and viability by MBCD. X-sperm had greater cell membrane damage than Y-sperm. These results may be due to differences in the cholesterol/phospholipid ratio between X- and Y-sperm membranes. Also this study was a new attempt to analyze the physiological difference between the X- and Y-sperm using flow cytometry.

## Methods

### Preparation of semen and MBCD

The semen was collected from ten healthy bulls of proven fertility (Hoengseong Livestock Cooperative Farm, Hoengseong, Korea) using an artificial vagina technique method (3 times per bull). All procedures that involved the use of animals were approved by the Kangwon National University Institutional Animal Care and Use Committee (KIACUC-09-0139). The ejaculated semen was transported to the laboratory at 25 °C within 1 h. The collected semen was diluted 1:1 (v/v) with the Triladyl extender (Minitüb, Tiefenbach, Germany). After maintenance at room temperature for 10 min, the semen was diluted to 5 × 10^7^ spermatozoa in 1 mL of Triladyl extender, and incubated with MBCD fresh solution (0, 1, 5, 10, and 20 mM) for 15 min at 25 °C. The incubated semen samples were centrifuged (400×*g*, 5 min) to remove MBCD from sperm before freezing.

### Freezing and thawing

Semen treated with MBCD was re-suspended with Triladyl extender containing 20 % egg yolk, and the semen cooled to 5 °C for 6 h. After cooling the semen was packaged into 0.5 mL straws cooled to −120 °C for 10 min before being plunged into liquid nitrogen for storage using static nitrogen vapor. For this study, the frozen sperms were thawed in a water bath at 37 °C for 45 s. The first prepared sperm samples were fluorescent-stained, and evaluated for viability and acrosome damage using flow cytometry (FACS Aria II, BD Biosciences, San Jose, CA, USA) and the staining patterns were verified by inspecting sperm samples under an epifluorescence microscope (Olympus, BX5, Tokyo, Japan). We used a dual blue/green filter set. The second prepared sperm samples were dyed with Hoechst 33342 and separated by the differential DNA content, the X and Y chromosomes, and then the viability and acrosome damage of sperm were analyzed.

### Sperm viability

The LIVE/DEAD^Ⓡ^ Sperm Viability Kit (L-7011, Invitrogen, Eugene, OR, USA) was used in the fluorescence-based assay method to analyze the viability of sperm. The sperms stained with SYBR-14 (live cell) and propidium iodide (PI, dead cell) and determined by flow cytometry. Briefly, the frozen-thawed semen samples washed in 1 mL phosphate buffered saline solution (PBS, 70,013, Invitrogen) and diluted semen samples in HEPES-buffered saline solution, and then treated with 5 μL of SYBR-14 dye. After the samples were incubated for 5–10 min at 37 °C, 5 μL of PI added into the 1 mL sample of diluted semen; then, the samples were incubated for 5–10 min at 37 °C, additionally. Finally, the semen samples were centrifuged at 400×*g* for 5 min in order to remove the dyes, and re-suspended in 1 mL PBS. 10,000 cells per sample were analyzed by a flow cytometer, 515–545 nm for SYBR-14 and 665–685 nm.

### Acrosome damage of sperm

The acrosome damage of sperm was assessed using fluorescein isothiocyanate-conjugated peanut agglutinin (FITC-PNA, L7381, Sigma-Aldrich, St. Louis, MO, USA). Briefly, the samples were stained with 50 ng mL^−1^ of FITC-PNA in 1 mL of frozen-thawed semen and incubated for 5–10 min at 37 °C. After incubation, the samples were centrifuged at 400×*g* for 5 min and re-suspended with 1 mL PBS. 10,000 cells per sample for acrosome damage of frozen-thawed bovine sperm were analyzed using a flow cytometer, 488 nm for positive, 515–545 nm for negative. The staining patterns could be verified by inspecting sperm samples under an epifluorescence microscope.

### Sex-sorting of spermatozoa

The frozen-thawed semen was stained with 40 μM Hoechst 33342 (B2261, Sigma-Aldrich, St. Louis, MO, USA) and incubated for 30 min at 37 °C in the dark [24]. Then, the incubated samples were stained with SYBR-14/PI for viability and FITC-PNA for acrosome damage. After staining with Hoechst 33342, SYBR-14/PI, the viability and acrosome damage of frozen-thawed sperm were analyzed to evaluate the effect of MBCD on X- and Y-bearing sperm of frozen-thawed semen. The Hoechst 33342-stained sperm was diluted with BTS containing 5 % egg yolk (v/v) and 0.002 % (w/v) food dye (FD&C no. 40, Warner Jenkinson Company Inc., St. Louis, MO, USA) before flow cytometry sorting. Especially, we used a food dye for selecting the viable sperm in sex sorting, because the reagent can penetrate cell membrane and quench fluorescent intensity of dead sperm. As shown in Fig. 3, the population of X- and Y-sperm was automatically sorted by Hoechst 33258, side-angle scatter (SSC), and forward angle scatter (FSC) using a FACS Aria II [25], and analyzed by flow cytometry for the viability and acrosome damage.

### Statistical analysis

The results were analyzed using SAS program (software version 9.1, SAS Institute Inc., Cary, NC, USA). Treatment groups were compared for differences using Duncan’s modified multiple range tests. Data were presented as mean ± SEM and a 5 % probability was considered significant.
